# Efficacy and tolerability of perampanel: a Chinese real-world observational study in epilepsy

**DOI:** 10.3389/fneur.2023.1286276

**Published:** 2024-01-19

**Authors:** Ya Zeng, Xintong Wu

**Affiliations:** ^1^Department of Pharmacy, West China Hospital, Sichuan University, Chengdu, China; ^2^Department of Neurology, West China Hospital, Sichuan University, Chengdu, China

**Keywords:** perampanel, real-world observational study, epilepsy, previous ASMs exposure, observational study

## Abstract

**Purpose:**

To investigate whether there exists a statistically significant distinction between the effectiveness and tolerance of perampanel (PER) and the number of antiseizure medications (ASMs) that were tried prior to administering PER.

**Method:**

A prospective, observational study was performed at West China Hospital of Sichuan University. The study included patients diagnosed with epilepsy who were prescribed PER and were monitored for a minimum of 6 months. The efficacy of PER was evaluated at 1, 3, 6, and 12-month intervals by examining the retention rate and the 50% response rate. All statistical analyses were conducted using IBM SPSS Statistics version 25 (IBM Corporation, Armonk, New York).

**Results:**

A total of 1,025 patients were identified, of which 836 were included in the analysis. Seven hundred and eighty-nine patients (94.4%) were followed up for a year. The median age of the patients was 29.32 ± 14.06 years, with 45.81% of the patients being male and 17.0% being adolescents. The average duration of epilepsy was 11.22 ± 8.93 years. Overall, PER was discontinued in 49.5% of patients, with the most common reasons being inadequate therapeutic effect and treatment-emergent adverse events (TEAEs). At the 6-month follow-up, the retention rate was 54.2% (454/836), and 39.6% of patients had a 50% response. At the 12-month follow-up, the retention rate was 49.4% (340/789), and 44.5% of patients had a 50% response. Patients who received PER as monotherapy had the highest retention rates (*P* = 0.034) and 50% response rates (*P* < 0.001) at any follow-up point. TEAEs were reported in 32.0% of patients, and these led to discontinuation in 15.4% of patients. The most common TEAEs were dizziness and somnolence. There was no significant difference between subgroups (*P* = 0.57), but there was a significant difference between the dosage of PER and TEAEs (*P* < 0.001).

**Main findings:**

The study concludes that PER is effective in treating both focal and generalized tonic-clonic seizures. Patients who had fewer previous exposures to ASMs exhibited higher response rates to PER. TEAEs related to PER dosage were more prevalent during the first 3 months of treatment and tended to improve with continued use, ultimately demonstrating favorable long-term tolerability.

## 1 Introduction

In the past decade, epilepsy has affected over 70 million people worldwide, making it one of the most common serious neurological disorders globally (1). In China, the prevalence and incidence rates are estimated to be 4.6–7.0 in 1,000 and 28.8–35.0 in 100,000, respectively (2, 3), and the disease places a significant strain on both the healthcare system and the affected individuals (4). Epilepsy patients often experience psychological or physical complications,^1^ a reduced quality of life (5), three times higher mortality than normal people (6), and increased costs for patients and social health insurance (7).

Antiseizure medications (ASMs) remain the primary treatment option for patients with epilepsy. Approximately 47% of patients with newly diagnosed epilepsy achieve seizure-free status after first monotherapy (8), and about 1/3 of these patients are refractory to the available drugs (9). And the proportion of drug-resistant patients does not appear to have changed over the previous 30 years (10), therefore, there is a need for new ASMs that can improve efficacy and tolerability of treatment.

Perampanel (PER) is the first and only non-competitive alpha-amino-3-hydroxyl-5-methyl-4-isoxazole-propionate (AMPA) receptor antagonist specifically engineered to block glutamate activity at postsynaptic AMPA receptors (11). PER is well-absorbed orally, has good plasma protein binding, and can be taken once daily, which can improve patient compliance. Furthermore, there is no significant interaction between PER and other ASMs.^2^ In the US, PER is approved as an adjunctive treatment and monotherapy for focal onset seizures (FOS) in patients aged ≥ 4 years and as an adjunctive treatment for generalized tonic-clonic seizures (GTCS) in patients aged ≥ 12 years (12). PER has also been approved in China and multiple other countries for treating both FOS with or without GTCS as adjunctive treatment and monotherapy (13). AS a new ASM characterized by non-competitive glutamate AMPA receptor antagonism, this peculiar mechanism of action is probably led to the role of PER in the bigger picture of ASM drugs, due to its optimal activity on myoclonic seizures (14), also justified by its neurophysiological profile affecting both cortical and sub-cortical paths (15, 16).

The efficacy and safety of adjunctive PER have been demonstrated in several randomized controlled trials (RCTs) and meta-analyses, include an analysis of phase III double-blind and open-label extension study in China (17–21). Some real-world retrospective studies conducted in Australia and Spain reported a 12-month retention rate of 55 and 80.5% at a medium maintenance dose of 6 mg/day, respectively, and identified several potential factors (gender, age, and previous ASMs) that may improve efficacy and tolerability, guiding the selection of the best PER treatment plan for patients (22, 23). in China, two previous observational studies reported 6-month retention rates of 77.8 and 67.9% at a mean maintenance dose of 4.96 ± 2.41 mg/day and 5.1 ± 1.5 mg/d, respectively (24, 25) one study reported 8-month retention rates of 72% at a mean maintenance dose of 5.9 ± 1.5 mg/day.

In this study, we conducted a 12-month prospective study in a real-world setting to systematically evaluate the efficacy and tolerability of PER in controlling FOS and GTCS as adjunctive treatment and monotherapy in Chinese patients with epilepsy. Our study included patients with any epilepsy type who had been taking PER for at least 4 years and who were receiving PER as monotherapy or in combination with any other therapeutic strategies. We hope that our study will provide useful data for the clinical application of PER in Chinese patients with epilepsy in the future.

## 2 Methods

### 2.1 Selection and description of participants

This study included patients diagnosed with epilepsy who were prescribed PER based on their clinician's recommendation from 2019 to 2022 at West China Hospital, Sichuan University. Patients were included if they met the following criteria: (1) confirmed diagnosis of epilepsy; (2) age ≥ 4 years; and (3) prescription of PER. Patients were excluded if they received PER for < 6 months. Study termination criteria: (1) patients who requested to withdraw from the study; (2) patients could not tolerate PER even with 2 mg/d; (3) pregnancy; (4) withdrawal due to other reasons.

All data were collected from our databases, and all patients were followed up by clinic visits or phone calls. The data collected included gender, age, seizure onset history, epilepsy etiology, epilepsy type, current and previous use of ASMs, PER titration and maintenance dosage, seizure frequency data from seizure diaries (or investigator assessment of therapeutic response if diaries were unavailable), and treatment-emergent adverse events (TEAEs) leading to withdrawal of PER (12). The study was reviewed and approved by the West China Hospital Medical Ethics Committee. The study was conducted in accordance with the principles of the Declaration of Helsinki. All patients or their legal guardian/next of kin gave written informed consent to participate in this study before enrollment.

### 2.2 Technical information

**Figure 1:**
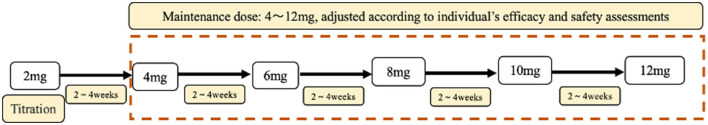
The titration and maintenance of PER. All patients were accepted standard treatment. Starting with 2 mg PER, PER dose titration occurred every 2 weeks. Until the optimal maintenance dose is reached. The maintenance dose was 4–12 mg.

In this study, patients with epilepsy were classified into five subgroups based on the number of previous ASMs they had been exposed to prior to taking PER (Table 1), regardless of which ASMs they were. PER was administered orally at a starting dose of 2 mg once daily at night and gradually increased by 2 mg every 2–4 weeks, with further increases at higher doses based on individual epileptologists' preferences, taking into consideration the dose-related risk of falls (20). Patients were followed up every 2–6 months after starting PER treatment. The “titration-phase” was typically set at 2–3 months, and outcomes were assessed thereafter (Figure 1).

**Table 1:** The definition of each subgroup.

**Subgroup**	**Define**
Monotherapy	Patients who didn't take any ASM before, and PER was the first ASM for them
First adjunctive ASM	Patients who had previously received only 1 ASM monotherapy regimen and PER was the second ASM for them
Second adjunctive ASM	Patients who had received 2 ASMs monotherapy or combined treatment, and PER was the third ASM for them
Third adjunctive ASM	Patients who had received 3 ASMs monotherapy or combined treatment, and PER was the forth ASM for them
Above third adjunctive ASM	Patients who had received > 3 ASMs monotherapy or combined treatment

The primary endpoint was the retention rate, defined as the proportion of the initial number of patients who remained on PER at 1, 3, 6, and 12 month follow-ups. The secondary efficacy endpoint were 50% response and seizure free rate, defined as the proportion of patients with a ≥50% reduction in seizure frequency or seizure free at 1, 3, 6, and 12 month follow-ups compared to the baseline seizure frequency on PER, including patients with seizure free since the previous follow-up.

Throughout the follow-up period, TEAEs were recorded and classified by severity as mild, moderate (requiring dose reduction or discontinuation), or severe (requiring medical treatment or resulting in death). The symptoms of each TEAE were also recorded.

### 2.3 Statistics

Sample size needed for the primary endpoint (the retention rate) in this study was calculated based on a multicenter, retrospective, non-interventional, Phase IV study that assess retention, efficacy, safety, and dosing of PER in patients with epilepsy during routine clinical care (12). It found that 24-month retention rates were 53.5% (*n* = 91/170) in adolescents and 47.8% (*n* = 354/741) in adults. If the lower limit of the 95% confidential interval (CI) of 24-month retention rates in our study was to be >40%, a sample size of ≥654 patients are needed to achieve a statistical power of 90%. Anticipating a dropout rate of 20%, a minimum of 785 patients were needed.

Missing data were not imputed, and all analyses were conducted using available data and performed using IBM SPSS Statistics version 25 (IBM Corporation, Armonk, New York). Qualitative variables were expressed as *n* (%), while quantitative variables were presented as mean ± SD. Student *t*-Tests or the non-parametric Mann–Whitney *U*-Test was used for comparing each subgroup for continuous variables, while Chi-squared tests or Fisher's exact test was used for categorical variables. Retention rate was assessed with counting the number of patients taking PER every month using the Kaplan–Meier survival analysis. All statistical analyses were conducted against a two-sided alternative hypothesis with a *p*-value < 0.05 considered to be statistically significant. Variables with a *p*-value of < 0.05 were included in the backward stepwise logistic regression analysis and presented as odds ratios (ORs) with 95% confidence intervals (CIs) and corresponding *p*-values.

## 3 Results

### 3.1 Patients (*n* = 836)

Initially, 1,025 patients were included in this study, but only 836 patients were analyzed due to 190 patients being lost to follow-up or having incomplete clinical data. The median age of the included patients was 29.32 ± 14.06 years, with 392 (46.9%) being male and 142 (17.0%) being adolescents. The mean duration of epilepsy was 11.22 ± 8.93 years. Patients who received PER as monotherapy had a shorter mean duration of epilepsy (4.92 ± 9.51 years) compared to those who received PER as adjunctive therapy (9.78 ± 8.40 years for first adjunctive therapy, 11.22 ± 8.80 years for second adjunctive ASM, 11.96 ± 9.71 years for third adjunctive ASM, and 10.82 ± 7.78 years for above third adjunctive ASM) (Table 2).

**Table 2:** Clinical characteristics of patients using PER (*n* = 836).

**Characteristics**	**Total (*n* = 836)**	**Monotherapy (*n* = 35)**	**First adjunctive ASM (*n* = 98)**	**Second adjunctive ASM (*n* = 432)**	**Third adjunctive ASM (*n* = 199)**	**>Third adjunctive ASM (*n* = 72)**
Male, *n* (%)	392 (46.9%)	17 (48.6%)	45 (45.9%)	208 (48.1%)	86 (43.2%)	34 (47.2%)
Adolescent, *n* (%)	142 (17.0%)	13 (37.1%)	18 (18.4%)	82 (19.0%)	22 (11.6%)	7 (9.7%)
Age at PER initiation (mean years)	29.32 ± 14.06	29.18 ± 17.19	28.82 ± 14.82	29.59 ± 13.90	30.63 ± 13.39	29.60 ± 14.09
Duration of epilepsy (mean years)	11.13 ± 9.41	4.92 ± 9.51	9.78 ± 8.40	11.22 ± 8.80	11.96+9.71	10.82+7.78
**Type of epilepsy**, ***n*** **(%)**						
Focal	331 (37.2%)	14 (40.0%)	38 (38.8%)	164 (40.0%)	71 (35.7%)	24 (33.3%)
Generalized	385 (46%)	14 (40.0%)	40 (40.8%)	198 (45.8%)	95 (47.7%)	38 (52.8%)
Epilepsy syndrome	54 (6.5%)	2 (5.7%)	9 (9.2%)	28 (6.5%)	13 (6.5%)	2 (2.8%)
Unclassified	86 (10.3%)	5 (14.3%)	11 (11.2%)	42 (9.7%)	20 (10.1%)	8 (11.1%)
**Etiology**, ***n*** **(%)**						
Known	338 (40.4)	14 (40.0%)	36 (36.7%)	168 (38.9%)	79 (39.7%)	41 (41.8%)
Assumed genetic	117 (14.0%)	4 (11.4%)	14 (14.3%)	60 (13.9%)	27 (13.6%)	12 (12.2%)
Trauma	8 (1.0%)	0 (0.0%)	2 (2.0%)	4 (0.9%)	2 (1.0%)	0 (0.0%)
Tumor	67 (8.0%)	3 (8.6%)	7 (7.1%)	33 (7.6%)	15 (7.5%)	9 (9.2%)
CNS infection	81 (10.0%)	4 (11.4%)	8 (8.2%)	39 (9.0%)	19 (9.5%)	11 (11.2%)
Other	65 (7.8%)	3 (8.6%)	5 (5.1%)	32 (7.4%)	16 (8.0%)	9 (9.2%)
Unknown	497 (59.6%)	21 (60.0%)	62 (63.3%)	264 (61.1%)	120 (60.3%)	31 (31.6%)
**Dose (mg/d)**, ***n*** **(%)**						
2 mg	47 (5.6%)	1 (2.9%)	6 (6.1%)	21 (4.9%)	13 (6.5%)	6 (8.3%)
4 mg	473 (56.6%)	11 (31.4%)	68 (69.4%)	252 (68.5%)	106 (61.8%)	36 (61.8%)
6 mg	174 (20.8%)	6 (17.1%)	11 (11.2%)	98 (18.1%)	40 (20.1%)	19 (26.4%)
8 mg	85 (10.2%)	12 (37.1%)	12 (12.2%)	29 (6.7%)	27 (8.5%)	5 (6.9%)
10 mg	38 (4.5%)	4 (5.7%)	1 (1.0%)	21 (1.9%)	8 (2.5%)	4 (1.4%)
12 mg	19 (2.3%)	1 (5.7%)	0 (0.0%)	11 (0.0%)	4 (0.5%)	2 (0.0%)
**Discontinuation from PER**, ***n*** **(%)**						
TEAEs	128 (15.3%)	5 (14.3%)	14 (14.3%)	65 (15.0%)	32 (16.1%)	12 (16.7%)
Inadequate therapeutic effect	154 (18.4%)	4 (11.4%)	10 (10.2%)	73 (16.0%)	52 (26.1%)	15 (20.8%)
Patient choice	80 (9.6%)	2 (5.7%)	7 (7.1%)	42 (9.7%)	20 (10.1%)	9 (12.5%)
Other	52 (6.2%)	1 (2.9%)	6 (6.1%)	23 (5.3%)	14 (7.0%)	8 (11.1%)

Among the patients, 37.2% (331) had focal epilepsy, 46% (385) had generalized epilepsy, 6.5% (54) had epilepsy syndrome, and 10.3% (86) had unclassified epilepsy. Of the patients, 40.4% (338) had identified epilepsy etiology, with genetic factors (14.0%) and central nervous system (CNS) infections (10.0%) being the most common causes. Other causes were trauma (1.0%), tumor (8.0%), and other (7.8%) (Table 2).

**Figure 2:**
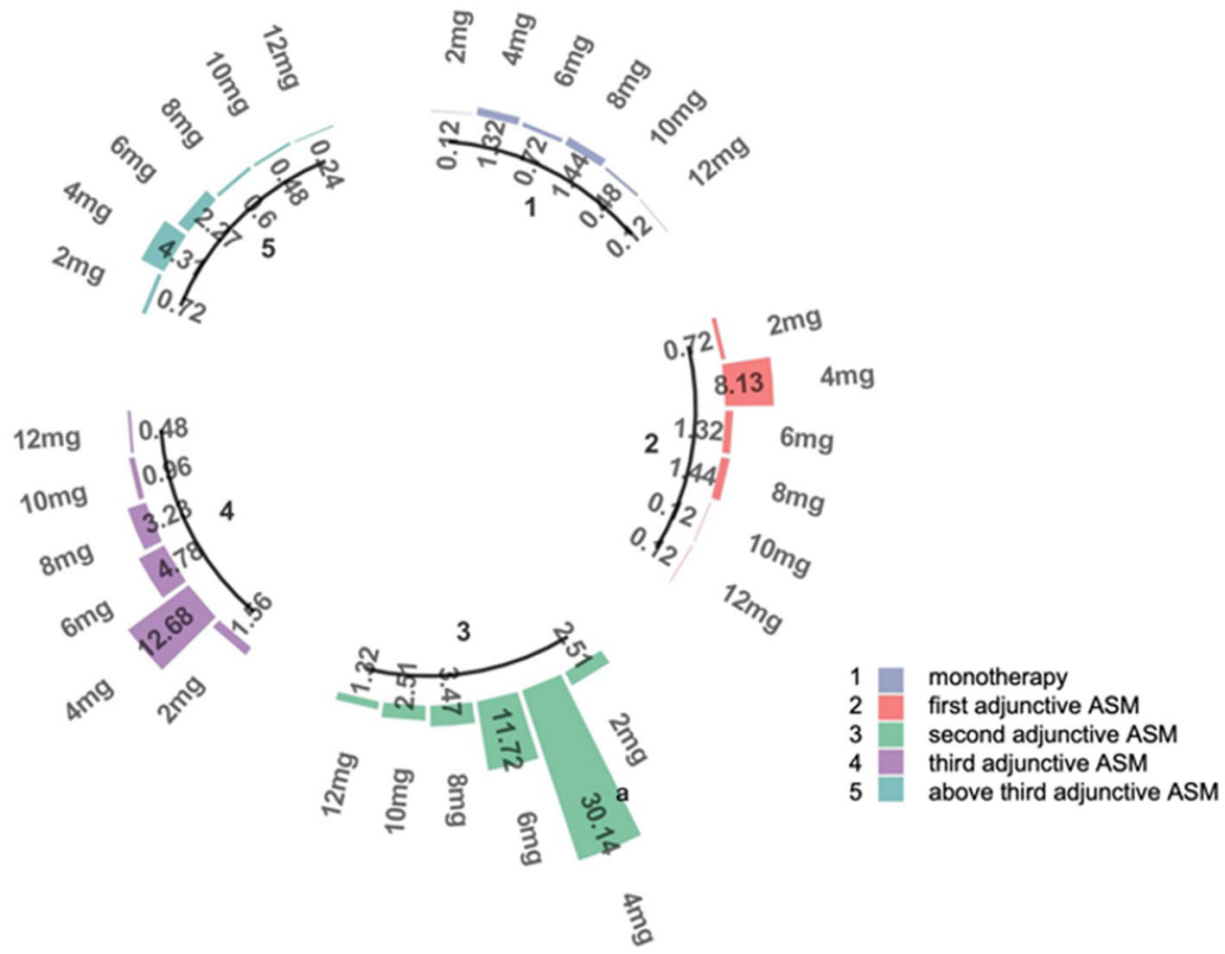
The doses of PER. This figure represents the proportion of patients receiving different doses in subgroups 1–5. 1: patients use PER as monotherapy; 2: patients use PER as first adjunctive therapy; 3: patients use PER as second adjunctive therapy; 4: patients use PER as third adjunctive therapy; 5: patients use PER as above third adjunctive therapy. a: The percentage of patients receiving 4 mg in subgroup 3, which accounts for 30.14% of the total number of patients.

The recommended maintenance dose of PER according to the instructions is 4–12 mg/day. In the whole study, the daily maintenance doses of PER were ordered as follows: 4 mg (64.5%), 6 mg/day (18.4%), 8 mg/day (9.1%), 2 mg (5.6%), 10 mg (2.0%), and 12 mg (0.4%) (Figure 2).

PER was discontinued in 414 (49.52%) patients after 1 year of follow-up. The most common reason for discontinuation was inadequate therapeutic effect (meaning that the seizure frequency did not change or became worse) (18.42%). Other reasons included TEAEs (15.31%), patient choice (usually related to economic factors or inconvenient purchase), and other reasons. The primary reason for discontinuation was slightly different in subgroups, but the difference was not significant (*P* = 0.11).

### 3.2 Efficacy (*n* = 836)

**Figure 3:**
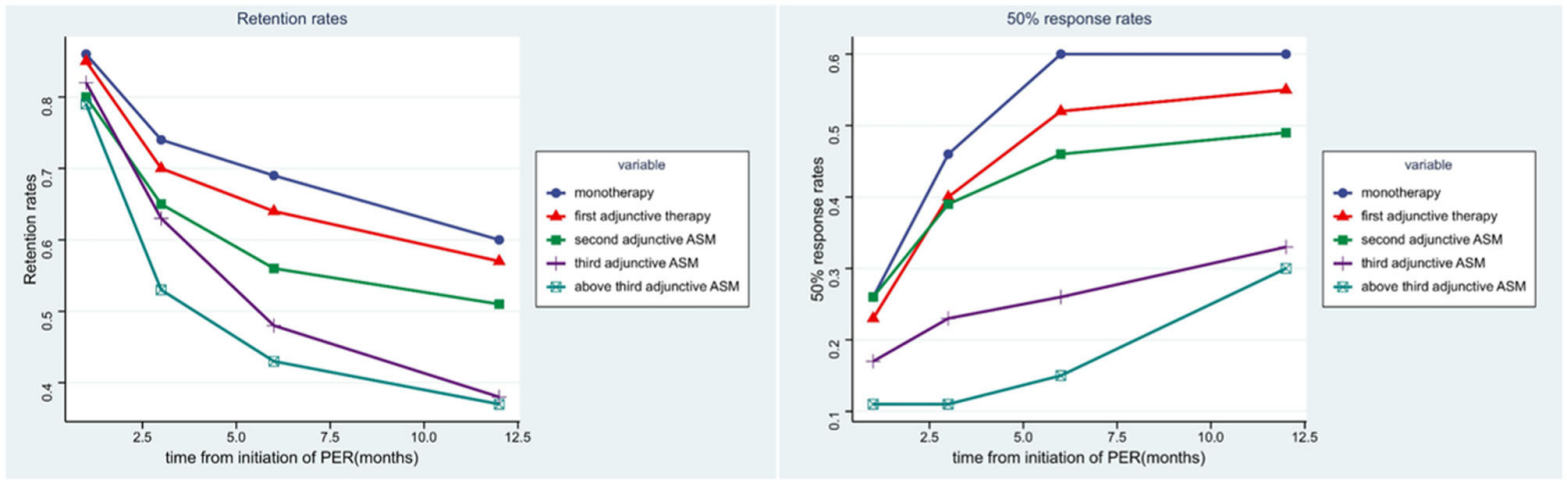
The efficacy of PER in five subgroups. The figure left represents the retention rates of each subgroups in 1, 3, 6, 12 months, the figure right represents the 50% response rates of each subgroups in 1, 3, 6, 12 months.

In our study,789 patients (94.38%) had been exposed to PER for over a year. Forty-seven patients (5.62%) were still undergoing PER treatment but had been exposed to PER for < 12 months, and they were excluded from the numerator and denominator at the 12-month follow-up. After 1 year of follow-up, approximately half of the patients (54.3%, 454/836) had achieved >6 months of exposure to PER (69% of patients received PER as monotherapy, 64% received PER as first adjunctive ASM, 56% received PER as second adjunctive ASM, 48% received PER as third adjunctive ASM, and 43% received PER as above third adjunctive ASM). About 49.4% (340/789) of the patients achieved >1 year of exposure (Table 3, Figure 3). Our analysis showed that patients who received PER as monotherapy had the highest retention rate, and the retention rate of PER decreased significantly with the number of previously failed ASMs (*P* = 0.034).

**Table 3:** The efficacy of PER.

**Characteristics**	**Total (*n* = 836)**	**Monotherapy (*n* = 35)**	**First adjunctive ASM (*n* = 98)**	**Second adjunctive ASM (*n* = 432)**	**Third adjunctive ASM (*n* = 199)**	**>Third adjunctive ASM (*n* = 72)**
**Retention rates**						
1 month	81.33%	85.71%	84.69%	80.09%	82.41%	79.17%
3 month	64.47%	74.29%	70.41%	65.05%	62.81%	52.78%
6 month	54.31%	68.57%	64.29%	55.56%	48.24%	43.06%
12 month	47.66%	60.00%	56.98%	51.09%	37.89%	37.14%
**50% response rates**						
1 month	22.24%	25.71%	23.47%	25.93%	17.09%	11.11%
3 month	32.25%	45.71%	39.80%	39.12%	23.12%	19.44%
6 month	39.59%	60.00%	52.04%	45.60%	25.63%	20.83%
12 month	44.49%	60.00%	54.65%	49.15%	32.63%	30.00%
**Seizure free**						
1 month	5.62%	14.29%	6.12%	7.18%	2.51%	0.00%
3 month	7.66%	17.14%	12.24%	9.37%	2.51%	1.39%
6 month	8.72%	20.00%	15.31%	9.72 %	3.52%	2.78%
12 month	19.68%	38.89%	30.61%	20.38%	9.72%	7.69%

The second efficacy outcome was the 50% response rate (which refers to the proportion of patients whose median percentage reduction in seizure frequency from baseline was >50%) and seizure free rate. Overall, after a month of PER treatment, the proportion of patients who had a 50% response rate and seizure free were 22.2 and 5.6%, respectively; 32.0 and 7.7% after 3 months of PER treatment; 40.0 and 8.7% after 6 months of PER treatment; 44.5 and 19.7% after 12 months of PER treatment. Seventy-four patients had been seizure free for the whole of 1 year (Table 3). It is evident that patients were most likely to have a 50% response rate in the first 6 months (Figure 3). Patients who received PER as monotherapy had the highest 50% response rate and seizure free rate, both rates decreased significantly with the number of previously failed ASMs before PER (*P* < 0.001).

**Figure 4:**
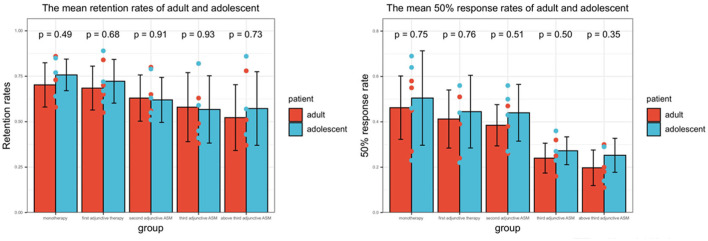
The efficacy of PER in adults and adolescents. The result represents the average value at time nodes 1, 3, 6, and 12 months. *P* < 0.05 represents there was significant difference between adults and adolescents.

The present analysis of effects included 142 (17.0%) adolescents (aged 4– < 18 years) and 694 (83.0%) adults (aged ≥ 18 years), with 11 adolescents and 36 adults not having 12-month follow-up data. Of the adolescents, 13 (37.1%) received PER as monotherapy. Approximately 55.6% of adolescents achieved > 6 months' exposure, and 52.7% achieved >12 months' exposure, while for adults, the corresponding figures were 54.0 and 46.7%, respectively. There were minor differences in retention rates and 50% response rates between adolescents and adults in all subgroups, but no significant differences were found (Figure 4).

In all types of epilepsy, FOS had the best 50% response, with 45.7% of patients after 6 months of PER treatment. GTCS had a response rate of 39.7%, the unclassified group had 31.3%, and patients with an epilepsy syndrome had the lowest response rate at 16.7%. The patient numbers in each group were not listed as the 50% response rates were not generally comparable across subgroups. No significant differences in 50% response rates were found between the different types of epilepsy (*P* = 0.065).

In all epilepsy etiologies, patients with other reasons had the best 50% response, with 44.6% of patients responding after 6 months of PER treatment. Patients with infection had a response rate of 42.0%, followed by those with an unknown reason at 40.0%, assumed genetic reason at 38.5%, trauma at 37.5%, and tumor had the worst response rate at 34.3%. No significant differences were found between the different epilepsy etiologies in terms of 50% response rates (*P* = 0.257).

PER was used as the first adjunctive ASM in 98 patients. The most commonly used concomitant ASMs were levetiracetam (LEV) (32, 32.65%), oxazepine (OXC) (27, 27.55%), valproate (VPA) (14, 14.29%), carbamazepine (CBZ) (8, 8.16%), and lamotrigine (LTG) (8, 8.16%). Patients who used CBZ (75.00%) and OXC (70.37%) before had the highest PER retention rate at the 6-month follow-up and remained high at the 12-month follow-up. They also had higher 50% response rates (Table 4). However, there were no significant differences between concomitant ASMs and retention rates (*P* = 0.635) or 50% response rates (*P* = 0.272).

**Table 4:** The most used concomitant ASMs in first adjunctive ASM group.

	**Retention rates**				**50% response rates**			
**Most used concomitant ASMs**	**1 month**	**3 month**	**6 month**	**12 month**	**1 month**	**3 month**	**6 month**	**12 month**
Levetiracetam (*n* = 32)	75.00%	65.63%	59.38%	53.13%	12.50%	31.25%	50.00%	53.13%
Oxazepine (*n* = 27)	88.89%	77.78%	70.37%	66.67%	29.63%	55.56%	66.67%	74.07%
Valproate (*n* = 14)	100.00%	71.43%	64.29%	64.29%	28.57%	42.86%	42.86%	57.14%
Lamotrigine (*n* = 8)	62.50%	62.50%	62.50%	62.50%	25.00%	25.00%	37.50%	50.00%
Carbamazepine (*n* = 8)	100.00%	75.00%	75.00%	75.00%	50.00%	50.00%	50.00%	62.50%
Topiramate (*n* = 6)	83.33%	83.33%	66.67%	66.67%	0.00%	33.33%	50.00%	50.00%
Clonazepam (*n* = 2)	100.00%	50.00%	50.00%	50.00%	50.00%	50.00%	50.00%	50.00%
Lacoxamine (*n* = 1)	100.00%	0.00%	0.00%	0.00%	0.00%	0.00%	0.00%	0.00%

### 3.3 Tolerability (*n* = 836)

TEAEs were reported in 267 (32.0%) patients (Table 5). The most common TEAEs were dizziness and somnolence (*n* = 194 [23.2%]), as well as aggression and irritability (*n* = 127 [15.2%]). The TEAEs were not significantly different between subgroups of patients stratified by the number of concomitant ASMs at baseline (*P* = 0.57). TEAEs leading to discontinuation were reported in 128 (15.4%) patients, with the most common ones being aggression and irritability (*n* = 77 [9.3%]) and dizziness and somnolence (*n* = 51 [6.1%]). Other TEAEs included weight gain, headaches, and diarrhea. No patients in our study required medical treatment or died from serious TEAEs related to PER. The dosage of PER was significantly associated with TEAEs and PER discontinuation due to TEAEs (*P* < 0.001).

**Table 5:** The TEAEs of PER.

**Patients type**	**Dosage**	**All patients**	**TEAEs, *n* (%)**	**TEAEs leading to PER discontinued, *n* (%)**
Monotherapy	4 mg	11	3 (27.27%)	0 (0)
	6 mg	6	2 (33.33%)	0 (0)
	8 mg	12	5 (41.67%)	1 (8.34%)
	10 mg	4	3 (75%)	2 (50)
	12 mg	1	1 (100%)	1 (100%)
First adjunctive ASM	4 mg	68	17 (25%)	7 (10.29%)
	6 mg	11	3 (27.27%)	1 (9.09%)
	8 mg	12	7 (58.33%)	4 (33.33%)
	10 mg	1	1 (100%)	1 (100%)
	12 mg	1	1 (100%)	1 (100%)
Second adjunctive ASM	4 mg	252	67 (26.59%)	35 (13.89%)
	6 mg	98	29 (29.59%)	11 (11.22%)
	8 mg	29	14 (48.28%)	5 (17.24%)
	10 mg	21	14 (66.67%)	8 (38.1%)
	12 mg	11	9 (81.82%)	6 (54.55%)
Third adjunctive ASM	4 mg	107	29 (27.1%)	10 (9.35%)
	6 mg	40	15 (37.5%)	8 (20%)
	8 mg	27	13 (48.15%)	9 (33.33%)
	10 mg	8	4 (50%)	3 (37.5%)
	12 mg	4	3 (75%)	2 (50%)
>Third adjunctive ASM	4 mg	36	11 (30.56%)	4 (11.11%)
	6 mg	19	9 (47.37%)	4 (21.05%)
	8 mg	5	3 (60%)	1 (20%)
	10 mg	4	3 (75%)	2 (50%)
	12 mg	2	1 (50%)	1 (50%)

## 4 Discussion

PER has been licensed for use as adjunctive therapy and monotherapy in patients with FOS, with or without GTCS, aged ≥ 4 years, and as adjunctive treatment of GTCS in patients aged ≥ 12 years, following sufficient RCTs to prove efficacy and safety (see text footnote 2). While RCTs may have favorable internal consistency, they may lack external application suitability, such as differences in drug dosage profiles from clinical practice. Epilepsy, as a chronic disease, requires real-world retrospective studies with longer follow-up times and larger sample sizes that can complement some of the limitations of RCTs. The American Academy of Neurology and International League Against Epilepsy Commission has recommended more meaningful long-term comparative trials that are representative of real-world clinical practices (26). Since 2016, the United States Congress has also approved the use of “real-world evidence” to replace traditional clinical trials to expand indications (12).

To date, PER is the only highly selective, noncompetitive AMPA glutamate receptor antagonist that is complementary to other ASMs on the market today (27). Patients in this study were divided into five subgroups based on the number of previously failed ASMs before PER as monotherapy, first adjunctive ASM, second adjunctive ASM, third adjunctive ASM, and above third adjunctive ASM. The study showed that PER was particularly successful for both FOS or GTCS patients, especially when used as the first option in patients who failed the first ASM, with a significant difference than more previous ASMs failed before. This result was in line with reported RCTs (28) and observational studies (29, 30) before. All these suggest that previous ASMs exposure may be associated with PER failure, no matter which ASMs they were. In this study, the use of fewer concomitant ASMs was associated with better outcomes. The reason may be related to drug-resistant epilepsy. The prognosis in newly diagnosed epilepsy is usually good, with up to 50% of people entering remission either without treatment or on their first ASM (31). A comparison of the effectiveness and tolerability of PER and brivaracetam shows no significant difference (32). Thus, the most powerful prognostic factor is the response of patients to the first ASM, not which ASM it is. This factor was particularly useful among patients in whom treatment at first or with the first ASM failed, PER did not show more effectiveness than other ASMs (32–34). we still need more studies to compare whether there are significant efficacy differences in different add-on ASMs, to provide evidence in the determination of which patients will benefit from different PER use.

An early response to drug therapy confers a favorable prognosis. In this study, the increase in the 50% response rate and seizure free rate were more significant in the first 6 months than in the last 6 months, especially in the first 3 months. The first 6 months are often referred to as the honeymoon period, especially in drug-resistant epilepsy (35).

Our study found that when PER was used as monotherapy, the proportion of adolescent was the highest, the efficacy is similar in adolescent and adults. But efficacy could not be objectively determined in this final analysis due to small adolescent numbers.

Efficacy did not differ significantly according to either epilepsy type or epilepsy etiology. PER was the first drug for treating FOS, so it had the largest number of studies to suggest its efficacy (4, 20, 36), and an observational study in Spain analyzed the effectiveness and tolerability of PER across different seizure types (30). PER can be useful for many different types of epilepsy, like idiopathic and genetic generalized epilepsy (37), brain tumor-related epilepsy (38), even status epilepticus (39). However, additional studies focusing on treated with PER for different epilepsy types and etiology remain necessary.

Drug interactions always lead to differences in the efficacy and tolerability of different ASM combinations. Margolis et al. described ASM combinations according to their mechanism of action and evaluated whether certain combinations affected efficacy or tolerability (40). We used their approach and assessed PER plus one ASM with a different mechanism of action and found no treatment advantage, even though OXC and VPA were found with higher retention rates and 50% response rates. However, in one study (30), patients previously treated with VPA had significantly higher retention rates of epilepsy than other ASMs.

From previous reports, the retention rates at 1 year were 70.4% (17), 53.5% in adolescents and 47.8% in adults (12), and 40.0% (22) from previous studies, while in our study it was 44.4%. The 50% response rate at 1 year was 65.8% (17), 79.3% in adolescents and 70.8% in adults (12), 21.7% (22), and 44.0% in our study. We analyzed the reasons for the large differences in results. Firstly, it might be due to differences in study design. There were 71 patients (8.5%) who were exposed to PER for < 12 months and were not included in the calculation in our study and the Australian study (22), This approach may lead to an underestimation of the retention rate at 12 months, like the intention-to-treat principle in RCTs. Secondly, there were differences in the studied populations, baseline characteristics, and the assessed drug regimens. Some previous studies had a majority of White patients (12, 22), FOS (4, 20, 36), and used PER as adjunctive ASM.

During this 1-year follow-up study, PER was generally found to be short-term and long-term safe and well-tolerated, similar to previous studies (12, 17, 22). PER can cause dose-related TEAEs like many other drugs. The most commonly reported TEAEs were dizziness, somnolence, fatigue, and irritability, similar to previous studies (12, 17, 22). Glutamate is one important neurotransmitter in the central nervous system (CNS) and may play a major role in epileptic activity, rapid eye movement (REM) sleep, and non-REM (41, 42). As the only AMPAR antagonist, these mechanisms of PER also lead to TEAEs in the CNS. TEAEs were reported in 32.0% of patients, which usually occur during the titration phase and tend to subside within a few weeks and decrease over time with the continuation of therapy or reduction of dose. Our study indirectly proved that PER tolerability is improved with low doses and a slow titration when PER is administered. TEAEs were reported leading to discontinuation in 15.4% of patients, which is less than regabalin in 46% of patients, zonisamide in 30% of patients, brivaracetam in 21% of patients, and 19% for LEV (33, 43). Some studies have shown that when PER is prescribed as the first adjunctive ASM and used with low doses ( ≤ 6 mg) and a slow titration (2 mg/day every >2 weeks), the frequency of TEAEs is halved (44, 45). In our study, 83.0% of patients in our study used PER with ≤ 6 mg, which may be the reason for fewer TEAEs discontinuations. The lack of efficacy, which was reported in 19.3% of patients, was the most commonly reported reason for PER treatment discontinuation.

Compared to most other ASMs, PER shows better compliance, allows for once-daily administration, and doesn't require monitoring of blood-drug concentration (27). Dizziness and somnolence can be reduced by taking PER at bedtime. Data about drug interactions with PER were limited, almost clinically insignificant, and no TEAEs caused by drug interactions have been reported to date (27).

The limitations of this study are as follows: First, like all retrospective studies, it is limited by inherent risks, including missing information, lack of randomization, potential for relevant information to be missing from records, patients' subjective records, and variations in follow-up timing (33). Secondly, only 17.82% of patients were adolescents, and we did not separate out subgroups of adolescent and elderly patients. Therefore, detailed information cannot be provided for these populations. Furthermore, we still need more comparative studies to identify predictors of long-term efficacy and provide strong guidance for clinicians to determine which patients will benefit from PER use (22).

## 5 Conclusions

This study confirms that the use of PER as monotherapy or adjunctive therapy was effective in controlling various types of epilepsy in a real-world setting and had long-term tolerability in patients. The high overall retention rate of 54.31 and 50% response rate of 44% underscored the good efficacy and tolerability of PER. Additionally, this study observed that patients who had more previous exposure to ASMs had lower response rates, which is consistent with a previous study on all ASMs.

## Data availability statement

The original contributions presented in the study are included in the article/Supplementary material, further inquiries can be directed to the corresponding author.

## Ethics statement

The studies involving humans were approved by West China Hospital, Sichuan University. The studies were conducted in accordance with the local legislation and institutional requirements. Written informed consent for participation in this study was provided by the participants' legal guardians/next of kin. Written informed consent was obtained from the individual (s), and minor(s)' legal guardian/next of kin, for the publication of any potentially identifiable images or data included in this article.

## Author contributions

YZ: Software, Writing – original draft. XW: Writing – review & editing.

## Footnotes

^1^*Epilepsy: a public health imperative*. Available onlin at: https://www.who.int/publications/i/item/epilepsy-a-public-health-imperative (accessed December 8, 2021).

^2^FYCOMPA^®^ Prescribing Information [Internet]. (2021).

## References

[1] Thijs RD Surges R O'Brien TJ Sander JW. Epilepsy in adults. Lancet. (2019) 393:689–701. 10.1016/S0140-6736(18)32596-0 30686584

[2] Trinka E Kwan P Lee B Dash A. Epilepsy in Asia: disease burden, management barriers, and challenges. Epilepsia. (2019) 60(Suppl. 1):7–21. 10.1111/epi.14458 29953579

[3] Trinka E Lee B. Epilepsy in Asia. Epilepsia. (2019) 60(Suppl. 1):5–6. 10.1111/epi.14507 30869166

[4] Zhao Y Atun R Oldenburg B McPake B Tang S Mercer SW. Physical multimorbidity, health service use, and catastrophic health expenditure by socioeconomic groups in China: an analysis of population-based panel data. Lancet Glob Health. (2020) 8:e840–9. 10.1016/S2214-109X(20)30127-3 32446349 PMC7241981

[5] Villanueva V Girón JM Martín J Hernández-Pastor LJ Lahuerta J Doz M. Quality of life and economic impact of refractory epilepsy in Spain: the ESPERA study. Neurologia. (2013) 28:195–204. 10.1016/j.nrleng.2012.04.013 22743210

[6] Callaghan B Choi H Schlesinger M Rodemer W Pollard J Hesdorffer DC. Increased mortality persists in an adult drug-resistant epilepsy prevalence cohort. J Neurol Neurosurg Psychiatry. (2014) 85:1084–90. 10.1136/jnnp-2013-307074 24554102

[7] Gao L Xia L Pan SQ Xiong T Li SC. Burden of epilepsy: a prevalence-based cost of illness study of direct, indirect and intangible costs for epilepsy. Epilepsy Res. (2015) 110:146–56. 10.1016/j.eplepsyres.2014.12.001 25616467

[8] Kwan P Brodie MJ. Early identification of refractory epilepsy. N Engl J Med. (2000) 342:314–9. 10.1056/NEJM200002033420503 10660394

[9] Lossius IMB Svendsen T Sødal HF Kjeldstadli K Lossius MI Nakken KO. Effect and tolerability of perampanel in patients with drug-resistant epilepsy. Epilepsy Behav. (2021) 119:107965. 10.1016/j.yebeh.2021.107965 33940525

[10] Chen Z Brodie MJ Liew D Kwan P. Treatment outcomes in patients with newly diagnosed epilepsy treated with established and new antiepileptic drugs: a 30-year longitudinal cohort study. JAMA Neurol. (2018) 75:279–86. 10.1001/jamaneurol.2017.3949 29279892 PMC5885858

[11] Lattanzi S Cagnetti C Foschi N Ciuffini R Osanni E Chiesa V. Adjunctive perampanel in older patients with epilepsy: a multicenter study of clinical practice. Drugs Aging. (2021) 38:603–10. 10.1007/s40266-021-00865-3 34075567 PMC8266697

[12] Segal E Wheless J Moretz K Penovich P Patten A Malhotra M. Perampanel in real-world clinical care of adolescent and adult patients with epilepsy: Results from the retrospective Phase IV PROVE Study. Seizure. (2022) 98:87–94. 10.1016/j.seizure.2022.02.011 35453064

[13] Tsai JJ Wu T Leung H Desudchit T Tiamkao S Lim KS. Perampanel, an AMPA receptor antagonist: from clinical research to practice in clinical settings. Acta Neurol Scand. (2018) 137:378–91. 10.1111/ane.12879 29214650

[14] Assenza G Nocerino C Tombini M Di Gennaro G D'Aniello A Verrotti A. Perampanel improves cortical myoclonus and disability in progressive myoclonic epilepsies: a case series and a systematic review of the literature. Front Neurol. (2021) 12:630366. 10.3389/fneur.2021.630366 33841303 PMC8024635

[15] Lanzone J Boscarino M Ricci L Insola A Tombini M Di Lazzaro V. Effects of the noncompetitive AMPA receptor antagonist perampanel on thalamo-cortical excitability: a study of high-frequency oscillations in somatosensory evoked potentials. Clin Neurophysiol. (2021) 132:1049–56. 10.1016/j.clinph.2020.12.030 33743300

[16] Lanzone J Ricci L Tombini M Boscarino M Mecarelli O Pulitano P. The effect of Perampanel on EEG spectral power and connectivity in patients with focal epilepsy. Clin Neurophysiol. (2021) 132:2176–83. 10.1016/j.clinph.2021.05.026 34284253

[17] Weiping L Dong Z Zhen H Patten A Dash A Malhotra M. Efficacy, safety, and tolerability of adjunctive perampanel in patients from China with focal seizures or generalized tonic-clonic seizures: Post hoc analysis of phase III double-blind and open-label extension studies. CNS Neurosci Ther. (2021) 27:330–40. 10.1111/cns.13458 33340263 PMC7871786

[18] French JA Krauss GL Steinhoff BJ Squillacote D Yang H Kumar D. Evaluation of adjunctive perampanel in patients with refractory partial-onset seizures: results of randomized global phase III study 305. Epilepsia. (2013) 54:117–25. 10.1111/j.1528-1167.2012.03638.x 22905857

[19] Nishida T Lee SK Inoue Y Saeki K Ishikawa K Kaneko S. Adjunctive perampanel in partial-onset seizures: Asia-Pacific, randomized phase III study. Acta Neurol Scand. (2018) 137:392–9. 10.1111/ane.12883 29250772

[20] Li Y Zeng Y Mu J Zhou D. The efficacy and safety of adjunctive perampanel for the treatment of refractory focal-onset seizures in patients with epilepsy: a meta-analysis. Epilepsia Open. (2022) 7:271–9. 10.1002/epi4.12574 34951748 PMC9159293

[21] Steinhoff BJ Patten A Williams B Malhotra M. Efficacy and safety of adjunctive perampanel 4 mg/d for the treatment of focal seizures: a pooled post hoc analysis of four randomized, double-blind, phase III studies. Epilepsia. (2020) 61:278–86. 10.1111/epi.16428 31944276 PMC7064985

[22] Sagar P Wawryk O Vogrin S Whitham E Kiley M Frasca J. Efficacy and tolerability of adjuvant perampanel: an Australian multicenter real-world observational study in refractory focal and generalized epilepsy syndromes. Epilepsy Behav. (2021) 119:107935. 10.1016/j.yebeh.2021.107935 33930626

[23] Abril Jaramillo J Estévez María JC Girón Úbeda JM Vega López Ó Calzado Rivas ME Pérez Díaz H. Corrigendum to “effectiveness and safety of perampanel as early add-on treatment in patients with epilepsy and focal seizures in the routine clinical practice: Spain prospective study (PERADON)” [Epilepsy Behav 102 (2020) 106655]. Epilepsy Behav. (2020) 104(Pt A):106917. 10.1016/j.yebeh.2020.106917 32005527

[24] Zhang R Qiao S Fang X Wang K Shi Y Du Q. Efficacy and tolerability of perampanel as adjunctive therapy in chinese patients with focal-onset seizures: an observational, prospective study. Front Neurol. (2021) 12:731566. 10.3389/fneur.2021.731566 34526963 PMC8435584

[25] Zhang Y Han X Zhao P Wang B Li M Zhao T. Perampanel add-on therapy for drug-refractory epilepsy: a single-center retrospective study based on 6-month treatment outcomes in Central China. Epilepsy Behav. (2022) 129:108617. 10.1016/j.yebeh.2022.108617 35219170

[26] Zeng QY Fan TT Zhu P He RQ Bao YX Zheng RY. Comparative long-term effectiveness of a monotherapy with five antiepileptic drugs for focal epilepsy in adult patients: a prospective cohort study. PLoS ONE. (2015) 10:e0131566. 10.1371/journal.pone.0131566 26147937 PMC4493091

[27] Bonanni P Gambardella A Tinuper P Acone B Perucca E Coppola G. Perampanel as first add-on antiseizure medication: Italian consensus clinical practice statements. BMC Neurol. (2021) 21:410. 10.1186/s12883-021-02450-y 34702211 PMC8549193

[28] Leppik IE Yang H Williams B Zhou S Fain R Patten A. Analysis of falls in patients with epilepsy enrolled in the perampanel phase III randomized double-blind studies. Epilepsia. (2017) 58:51–9. 10.1111/epi.13600 27869305

[29] Labate A Fortunato F Giugno A Martino I Caligiuri ME Gambardella A. Perampanel as first add-on choice on the treatment of mesial temporal lobe epilepsy: an observational real-life study. Neurol Sci. (2021) 42:1389–94. 10.1007/s10072-020-04636-7 32772278

[30] Villanueva V Montoya J Castillo A Mauri-Llerda J Giner P López-González FJ. Perampanel in routine clinical use in idiopathic generalized epilepsy: The 12-month GENERAL study. Epilepsia. (2018) 59:1740–52. 10.1111/epi.14522 30062784

[31] Brodie MJ. Antiepileptic drug therapy the story so far. Seizure. (2010) 19:650–5. 10.1016/j.seizure.2010.10.027 21075011

[32] Liguori C Manfredi N Renna R Izzi F Pagliuca M Pagliuca F. Comparison of the effectiveness and tolerability of perampanel and brivaracetam: a real-world, observational, retrospective study. Epileptic Disord. (2020) 22:309–16. 10.1684/epd.2020.1165 32540833

[33] Strzelczyk A Zaveta C von Podewils F Möddel G Langenbruch L Kovac S. Long-term efficacy, tolerability, and retention of brivaracetam in epilepsy treatment: a longitudinal multicenter study with up to 5 years of follow-up. Epilepsia. (2021) 62:2994–3004. 10.1111/epi.17087 34608628

[34] Hufnagel A Ben-Menachem E Gabbai AA Falcão A Almeida L Soares-da-Silva P. Long-term safety and efficacy of eslicarbazepine acetate as adjunctive therapy in the treatment of partial-onset seizures in adults with epilepsy: results of a 1-year open-label extension study. Epilepsy Res. (2013) 103:262–9. 10.1016/j.eplepsyres.2012.07.014 22871333

[35] Löscher W Poulter MO Padjen AL. Major targets and mechanisms of antiepileptic drugs and major reasons for failure. Adv Neurol. (2006) 97:417–27. 16383152

[36] Wang Q Xu Y Chen Y Wu X Ge Y Zhu G. Effectiveness and safety of perampanel as adjunctive therapy among Chinese patients with focal-onset epilepsy: A real-world prospective observational study. Epilepsy Behav. (2022) 136:108937. 10.1016/j.yebeh.2022.108937 36215830

[37] French JA Krauss GL Wechsler RT Wang XF DiVentura B Brandt C. Perampanel for tonic-clonic seizures in idiopathic generalized epilepsy A randomized trial. Neurology. (2015) 85:950–7. 10.1212/WNL.0000000000001930 26296511 PMC4567458

[38] Coppola A Zarabla A Maialetti A Villani V Koudriavtseva T Russo E. Perampanel confirms to be effective and well-tolerated as an add-on treatment in patients with brain tumor-related epilepsy (PERADET study). Front Neurol. (2020) 11:592. 10.3389/fneur.2020.00592 32695064 PMC7336340

[39] Trinka E Höfler J Leitinger M Brigo F. Pharmacotherapy for status epilepticus. Drugs. (2015) 75:1499–521. 10.1007/s40265-015-0454-2 26310189 PMC4559580

[40] Margolis JM Chu BC Wang ZJ Copher R Cavazos JE. Effectiveness of antiepileptic drug combination therapy for partial-onset seizures based on mechanisms of action. JAMA Neurol. (2014) 71:985–93. 10.1001/jamaneurol.2014.808 24911669

[41] Di Bonaventura C Labate A Maschio M Meletti S Russo E. AMPA receptors and perampanel behind selected epilepsies: current evidence and future perspectives. Expert Opin Pharmacother. (2017) 18:1751–64. 10.1080/14656566.2017.1392509 29023170

[42] Dash MB Douglas CL Vyazovskiy VV Cirelli C Tononi G. Long-term homeostasis of extracellular glutamate in the rat cerebral cortex across sleep and waking states. J Neurosci. (2009) 29:620–9. 10.1523/JNEUROSCI.5486-08.2009 19158289 PMC2770705

[43] Novy J Bartolini E Bell GS Duncan JS Sander JW. Long-term retention of lacosamide in a large cohort of people with medically refractory epilepsy: a single centre evaluation. Epilepsy Res. (2013) 106:250–6. 10.1016/j.eplepsyres.2013.05.002 23796862

[44] Abril Jaramillo J Estévez María JC Girón Úbeda JM Vega López Ó Calzado Rivas ME Pérez Díaz H. Effectiveness and safety of perampanel as early add-on treatment in patients with epilepsy and focal seizures in the routine clinical practice: Spain prospective study (PERADON). Epilepsy Behav. (2020) 102:106655. 10.1016/j.yebeh.2019.106655 31812902

[45] Santamarina E Bertol V Garayoa V García-Gomara MJ Garamendi-Ruiz I Giner P. Efficacy and tolerability of perampanel as a first add-on therapy with different anti-seizure drugs. Seizure. (2020) 83:48–56. 10.1016/j.seizure.2020.09.026 33096456

